# Diversity of mitochondrial genes and predominance of Clade B in different head lice populations in the northwest of Iran

**DOI:** 10.1186/s13071-020-04364-z

**Published:** 2020-09-23

**Authors:** Mohammad Bagher Ghavami, Maryam Ghanbari, Sanaz Panahi, Behrooz Taghiloo

**Affiliations:** Department of Medical Entomology and Vector Control, School of Medicine, Zanjan University of Medical Sciences, Zanjan, Iran

**Keywords:** Pediculosis, *Pediculus humanus capitis*, *cytb* gene, *cox*1 gene, Mitochondrial clade A, Mitochondrial clade B

## Abstract

**Background:**

The head louse, *Pediculus humanus capitis*, is the most important ectoparasite causing many health problems. Several linkages are presented for this parasite, each representing a particular geographical distribution, prevalence rate, vector competence, susceptibility to pediculicides, and infestation rate. Determining the genetic nature of these linkages is necessary to identify the population structure and also to develop and monitor control programmes against head lice. This study was designed to analyse *cox*1 and *cytb* genes and determine the mitochondrial clades in head lice populations in the northwest of Iran.

**Methods:**

Adult head lice were collected from infested females of Ardabil, East and West Azerbaijan, and Zanjan Provinces from 2016 to 2018. Partial fragments of the mitochondrial genes *cox*1 and *cytb* were amplified by PCR and some of the amplicons were sequenced. All confirmed sequences were analysed, and the frequency of each mitochondrial clade was determined in the studied areas.

**Results:**

A total of 6410 females were clinically examined, and 897 adult head lice were collected from 562 infested cases. Genomic DNA was extracted from 417 samples, and fragments of *cox*1 and c*ytb* genes were amplified in 348 individuals. Analysis of the 116 sequences showed the 632-bp and 495-bp fragments for *cox*1 and *cytb* genes, respectively. The nucleotide and haplotype diversities of *cytb* and *cox*1 genes were 0.02261 and 0.589 and 0.01443 and 0.424, respectively. Sequence analysis indicated 6 haplotypes clustered in two clades, A and B. The relative prevalence of clade B was 73% for *cytb* and 82% for *cox*1 gene. Haplotypes of clade B were found in all the studied areas, while those of clade A were observed only in rural and suburban areas.

**Conclusions:**

To our knowledge, this is the first study investigated deeply the field populations of *Pediculus* and documented two clades in the Middle East. The considerable prevalence of pediculosis in the studied areas requires authorities’ attention to establish effective control and preventive measures. Given the role of *cytb* in monitoring population groups, application of this marker is suggested for future epigenetic studies to evaluate the factors affecting the abundance of these clades.
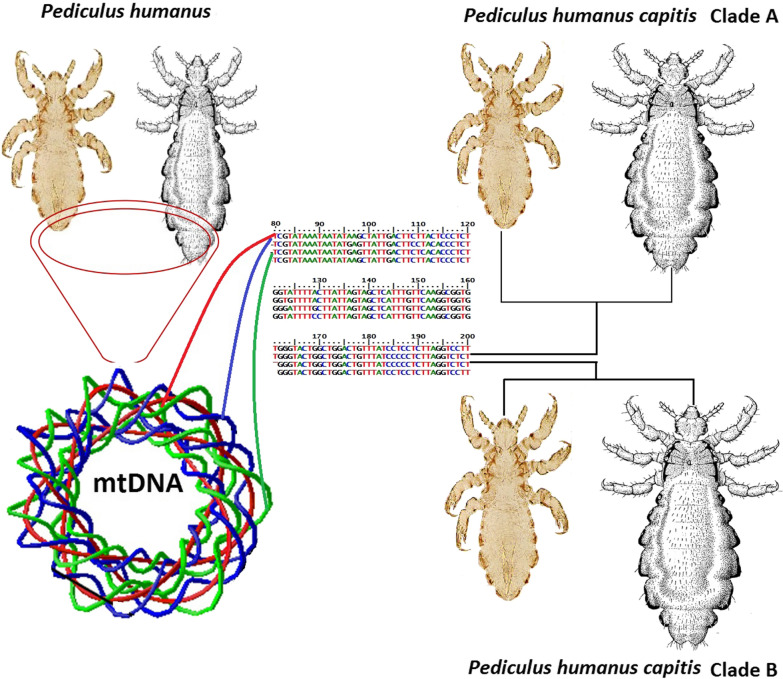

## Background

The head louse, *Pediculus humanus capitis* De Geer, is an important hematophagous ectoparasite of humans, and infestation with this obligate ectoparasite constitutes one of the most serious public health problems worldwide [1]. Dermatitis, fatigue, psychological irritability, paranoia, and weakness are the most common allergic reactions caused by pediculosis capitis. Other related complications include depression, hyperthermia, headache, feeling of heaviness in the body, insomnia, muscle rigidity, attention deficit in the class and educational failure, lack of social status, developing secondary infections, and hair loss [2, 3]. This louse has been associated with humans for thousand years and is dispersed throughout the world due to human migration [4, 5]. In spite of advances in health care and medical education in the third millennium, head lice infestation has dramatically increased in the past decades and threatened community health, particularly children between 5 and 13 years-old [3, 6].

The body louse, *P. humanus humanus* Linnaeus, is another subspecies of human louse, which seriously endangers public health and is often misdiagnosed as a head louse [7]. Besides, the epidemiological features of this subspecies are often different from those of the head louse. Today, the body louse is less common in developed nations and persists as a significant problem only in less developed countries. In recent years, the prevalence of this louse has declined and has been limited to individuals living in poor health conditions, refugees, and the homeless in urban areas. Although there are phenotypic differences between head and body lice in terms of body length and form of antennal and abdominal segments, some specimens of these subspecies are indistinguishable in appearance [8]. The main dissimilarities between the two subspecies are related to their population ecology, physiological adaptations, immunological responses, and vector competence [9]. Moreover, the response of hemocytes in typical head and body lice is comparable. In head lice, the hemocytes attack and engulf the symbionts almost instantly when they leave the stomach disc mycetome, but in body lice, the hemocyte response is delayed by several hours, and hemocytes with engulfed symbiont are rarely observed [10]. The body louse is an important vector for *Borrelia recurrentis*, *Bartonella quintana* and *Rickettsia prowazekii* [10–12]. Experimental infection with *Rickettsia rickettsii* (causing Rocky Mountain spotted fever) and *Rickettsia conorii* (causing Mediterranean spotted fever) have also been reported in this subspecies. However, the ability of head lice to transmit *R. prowazekii* has been indicated only in laboratory experiments, and evidence for such transmission in the field has not yet been reported [12, 13].

Female lice do not normally have spermathecae. This condition makes them mate repeatedly before laying eggs. In addition, the high mobility of lice is rarely recognized, and their immobility enhances their recombination frequency and genetic diversity [8]. On the other hand, the lack of a scheduled control programme, excessive use of over-the-counter pediculicides and broad-spectrum antibiotics, insecticide resistance, and host-parasite coevolution influence speciation, which result in the occurrence of varied biological forms in head louse populations [14].

In recent decades, molecular studies have revealed many facts about different groups of head lice populations. Recombination between 20 mini-circular chromosomes in lice mitochondria brings wide genomic plasticity in mtDNA fragments [15–19]. Interbreeding between different groups of lice has shown highly variable spacers in cytochrome *b* (*cytb*) [8]. Potential recombination events between these groups demonstrate that their evolution is not dichotomic, and their behaviour is very close to that of a rhizome [20]. Human lice can differentiate into six deeply divergent mitochondrial clades (A, B, C, D, E and F), each having a unique geographical distribution. Clades A and B are the most common and widely distributed throughout the world whereas, the other clades are geographically restricted. Clade C has been found in some countries of Africa and Asia. Clades D and E appear to be specific to African lice, and the last group, clade F, has been recovered from Amazonian lice. With the exception of two clades (B and C) comprising only head lice, the remaining groups (clades A, D, E and F), are comprised of both head and body lice [4, 5, 20–26]. Current phylogenetic and population genetic analyses of these mitochondrial lineages have indicated that clade A, the most recent group, more likely to have originated in Africa and initially migrated to Eurasia and then to the New World. This clade is divided into three main subclades: the Eurasian subclade A1; the sub-Saharan subclade A2; and the pre-Colombian subclade A3 [22, 27]. The exact source of clade B remains unknown. Its evolutionary origin might be found in the archaic hominids from Eurasia, and it may have become associated with modern humans *via* host switching during the period of overlap [5, 24, 25].

Various prevalence levels of pediculosis have been reported globally during the past two decades [2, 6, 14, 28–30]. Recent studies on pediculosis have revealed that among Asian countries, the number of studies are overrepresented in Turkey and Iran. The prevalence of head lice infestation varies from 0.3% to 42.6% in these countries [31–35]. Such significant variation in pediculosis has also been observed in various provinces of Iran. The findings of these different studies and the results of systematic reviews and meta-analytic methods have suggested that the prevalence (95% confidence interval (95% CI)) of head lice infestation among Iranian primary school children is estimated as 1.6% (95% CI: 1.2–2.05), 8.8% (95% CI: 7.6–9.9), and 7.4% (95% CI: 6.6–8.2) among boys, girls, and all the students, respectively [31]. Numerous studies have reported a remarkably high prevalence of head lice among females in the northwest of Iran [36–40]. Evidence has proven that risk factors such as occupation, combing hair per day, bathing per week, itching sensation, family size, and presence or absence of health conditions contribute to the prevalence of head lice. Access to health services, potential introduction of effective pediculicides, history of applied pediculicides, and genotype of parasite strains are other factors that play a role in the overall prevalence of pediculosis in the country. Based on socioeconomical, biological, operational and technical impact factors, the prevalence of pediculosis varies in three settlement types in Iran, urban, suburban and rural [31, 41].

Understanding genetic diversity in head lice populations is crucial for analysing the spread of the lice-borne diseases in human populations and for designing essential preventive control methods against these diseases. This knowledge is also significant for use in basic and evolutionary biology and also for defining the mechanisms that maintain the parasite within host populations. Given the wide genomic plasticity of lice mitochondria, the study of *cytb* and cytochrome *c* oxidase subunit 1 (*cox*1) genes, as molecular markers, could represent important information about the evolutionary processes of parasites in different populations.

In view of the lack of genetic studies on head lice in Iran and the very scant and limited surveys on this subject, we determined the relative frequency, geographical distribution, and genetic diversity of the mitochondrial clades of head lice collected from different geographical localities (urban, suburban and rural areas) in the northwest of Iran. *Cytb* and *cox*1 mtDNAs were also employed to determine the genotypes of head lice.

The information presented in this study is helpful for understanding the population structure and epidemiology of lice, in order to strengthen further efforts to prevent the re-emergence of the disease and to achieve more data on developing and monitoring head lice control programmes. This information would also update our knowledge of the diversity of head lice in the Middle East and contribute to the understanding of human and louse co-evolution.

## Methods

### Collection of head louse specimens

Adult head lice were collected from the head of infested females in 13 districts of East Azerbaijan, West Azerbaijan, Ardabil, and Zanjan Provinces, in the northwest of Iran, from 2016 through 2018 (Table 1). The participants of the study included elementary students (6–15 years-old) in 52 schools and women who visited six local medical centres to receive services. In this study, volunteers who felt itchy in both the scalp and neck were selected for clinical examination. Temporal and post-occipital areas of head were examined for the presence of lice or nits. After examination, the volunteers wore a disposable white apron, their hair was combed with a metal head lice comb, and the collected lice samples in the apron were transferred to vials containing 70% ethanol and kept at − 20 °C for further analysis. The epidemiological data, residence address (rural, suburban and urban areas), and clinical data (infestation rate) were recorded, and following the lice collection, all participants in the study received permethrin shampoo.

**Table 1:** Locality of study areas and frequency of collected head lice from various settlement types

Province	District	Geographical coordinates		Type of settlement			Total
		Latitude (N)	Longitude (E)	Urban	Suburban	Rural	
Ardabile	Ardabile	38.1201–38.5163	48.1294–48.3703	10	17	15	42
	Khalkhal	37.6111–37.8753	48.2770–48.3936	8	16	12	36
East Azarbayjan	Bostan Abad	37.7933–37.8578	46.8483–46.8503	6	4	12	22
	Myianeh	37.2126–37.5226	47.4541–47.9954	64	67	150	281
	Tabriz	37.9363–38.3294	45.9187–46.3368	67	74	45	186
	Varzqan	38.4912–38.5116	46.6126–46.6821	8	4	8	20
West Azarbayjan	Khoy	38.5715–38.5895	44.9051–45.0390	7	25	35	67
	Salmas	38.1913–38.3400	44.6926–44.8450	4	12	15	31
	Tekab	36.4119–36.6062	46.9483–47.1116	4	3	5	12
	Urmia	37.5308–38.1765	44.1905–45.2080	14	15	17	46
Zanjan	Khodabandeh	35.8425–36.2536	47.6499–48.7449	5	6	3	14
	Mahneshan	36.7390–36.8799	47.6704–47.6880	15	10	35	60
	Zanjan	36.6367–36.6881	48.5214–48.5844	36	20	24	80
Total				248	273	376	897

### Genomic DNA (gDNA) extraction

Lice were washed with ddH_2_O and stored at − 70 °C for 2 h. The frozen lice were placed individually in a sterile glass Petri dish and chopped in a 400 µl lysis buffer (100 mM of Tris HCl (*p*H 8.0), 0.5 mM of NaCl, 10 mM of EDTA and 1% W/V SDS). The samples were transferred to 1.5 ml tubes and homogenized with glass beads by an electric homogenizer. Subsequently, 20 µg of proteinase K was added to the homogenates before incubation at 55 °C for 3 h. Next, 100 µl of 8 M potassium acetate was added to each tube, mixed gently and kept on ice bath for 30 min. The samples were centrifuged at 4000×*g* for 10 min, and the supernatants were transferred to fresh tubes. One ml of ice-cold absolute ethanol was added to each tube, followed by centrifugation at 8000×*g* for 10 min. Pellets were washed in 0.5 ml of 70% ethanol, before centrifugation at 8000×*g* for 10 min. The final pellet was dried at room temperature and re-suspended in 100 µl of TE buffer.

### Allele-specific amplifications of mitochondrial genes

The amplification reaction for mitochondrial genes were set up in a final reaction volume of 50 µl, containing 10 µl of gDNA (approximately 50 ng), 10 pM of *cox*1: POF (5′-ATA GTT ATG CCT GTA ATA ATA G-3′) and POR (5′-TGT TGG TAT AAA ACA GGA TCA C-3′) and *cytb*: PBF (5′-GAA ATT TTG GGT CTT ATT AGG-3′) and PBR (5′-TCA ACA AAA TTA TCC GGG TC-3′) primers, and 25 µl of 2× Red Master Mix (Ampliqon, Odense, Denmark). The universal (PBR and POR) primers were used as reverse primers in previous investigations [21, 42, 43]. However, the specific forward (PBF and POF) primers were designed from the conserved region of 5’ end of reference sequences on GenBank (EU93439, AY695942, AY696013 and AY696047). PCR was performed by one cycle of initial denaturation at 95 °C for 5 min, followed by 35 cycles, each consisting of 95 °C for 30 s, 52 °C for 30 s, and 72 °C for 90 s, and a final extension step at 72 °C for 7 min. Five µl of each PCR product was assayed by electrophoresis on a 1.5% agarose gel at 80 V for 45 min and visualized under UV light.

### Analysis of PCR products

The amplified fragments were purified using a PCR Clean-Up Kit (SinaClon, Tehran, Iran) and sequenced bi-directionally by Macrogen (Seoul, Korea) using the forward and reverse specific primers. The nucleotide sequences of samples were edited using BioEdit version 7 software. All sequences were aligned with Clustal Omega and edited manually to check indels and single nucleotide polymorphisms within homologous groups. The nucleotide sequences were translated into amino acid sequences of proteins. Sequences trace files were analysed with MEGA X [44], DNAStar 6 [45] and PopART version Qt4.8.4 (http://popart.otago.ac.nz) software, used for constructing phylogenetic trees and identifying haplotype groups and nucleotide diversity. The resulting alignments were checked by eye, and phylogenetic relationships were performed using Neighbor-Joining and Maximum Likelihood analysis in MEGA X using the Kimura 2-parameter model for nucleotide sequences under 500 bootstrap replicates. The alignments of the studied gene sequences were also used to construct haplotype networks using the TCS method [46]. Finally, the alignments were checked against *cox*1 and *cytb* reference sequences available on GenBank; the chimpanzee louse, *P. schaeffi*, was selected as the outgroup [47].

## Results

Of 6410 females enrolled in this study, 562 girls (8.7%) were infested with *P. humanus capitis*. Of these, 154 (8.3% of 1859), 172 (8.9% of 1923) and 236 (9.0% of 2628) girls were from the urban, suburban and rural areas, respectively. Of 897 adult head lice collected from infested girls, 248 (27.65%) were from urban, 273 (30.43%) from suburban, and 376 (41.92%) from rural areas (Table 1). DNA was extracted from 417 randomly selected head lice (12–36 samples from each district). The concentration of nucleic acids in the studied samples was within the range of 260.5–920.8 ng/µl. The ratio of absorbance at 260 nm and 280 nm (260/280 ratio) and 230/260 ratio were within the range of 1.59–2.89 and 1.0–2.29, respectively. Partial fragments of the mitochondrial genes *cytb* and *cox*1 were amplified for 348 individuals, and 116 samples were randomly selected for sequencing.

**Fig. 1:**
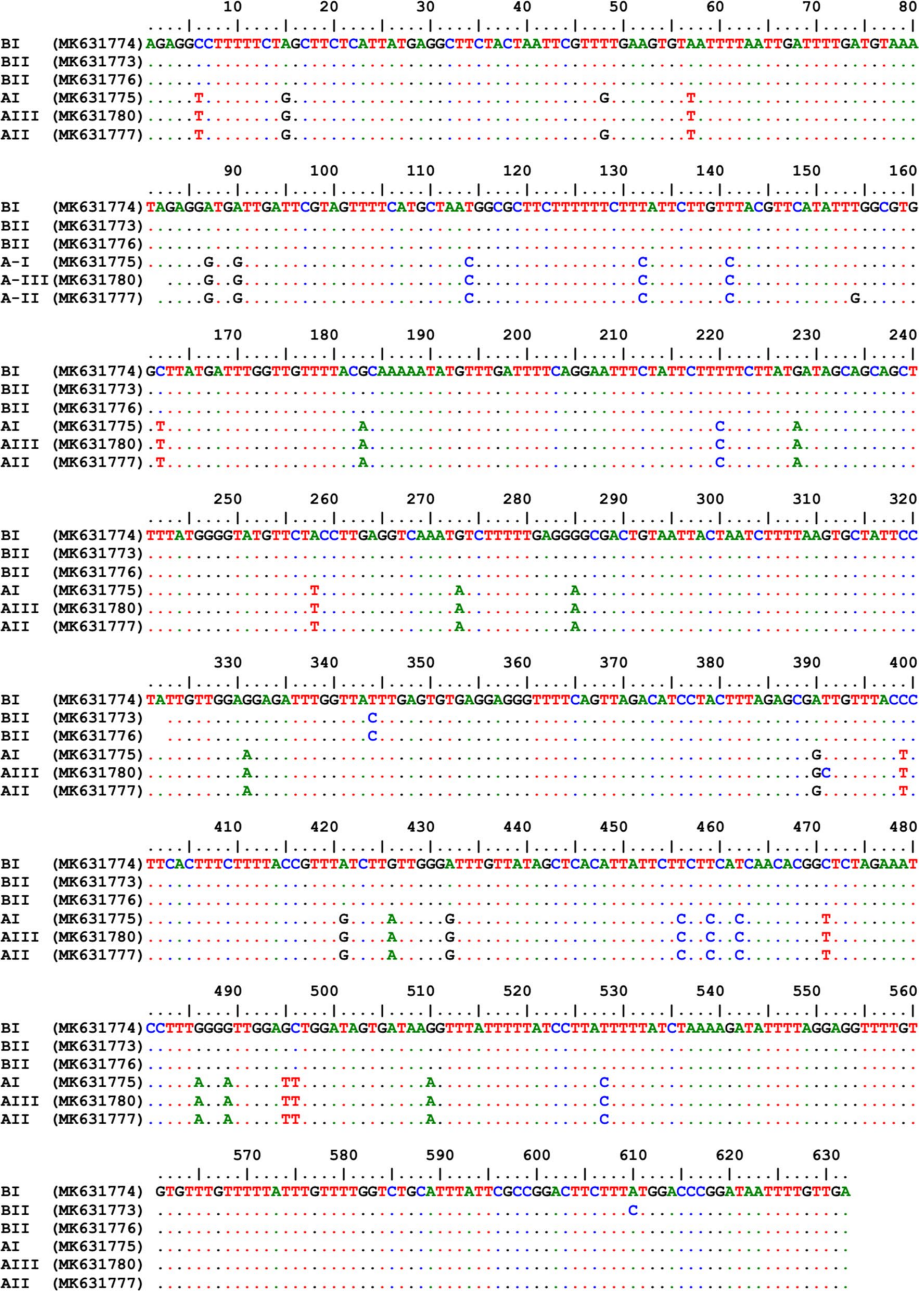
Alignment of the nucleotide sequences of the mitochondrial *cytb* gene fragment in different haplotypes in head lice populations in the northwest of Iran. The numbers in parentheses are the representative accession numbers of the haplotype groups

**Fig. 2:**
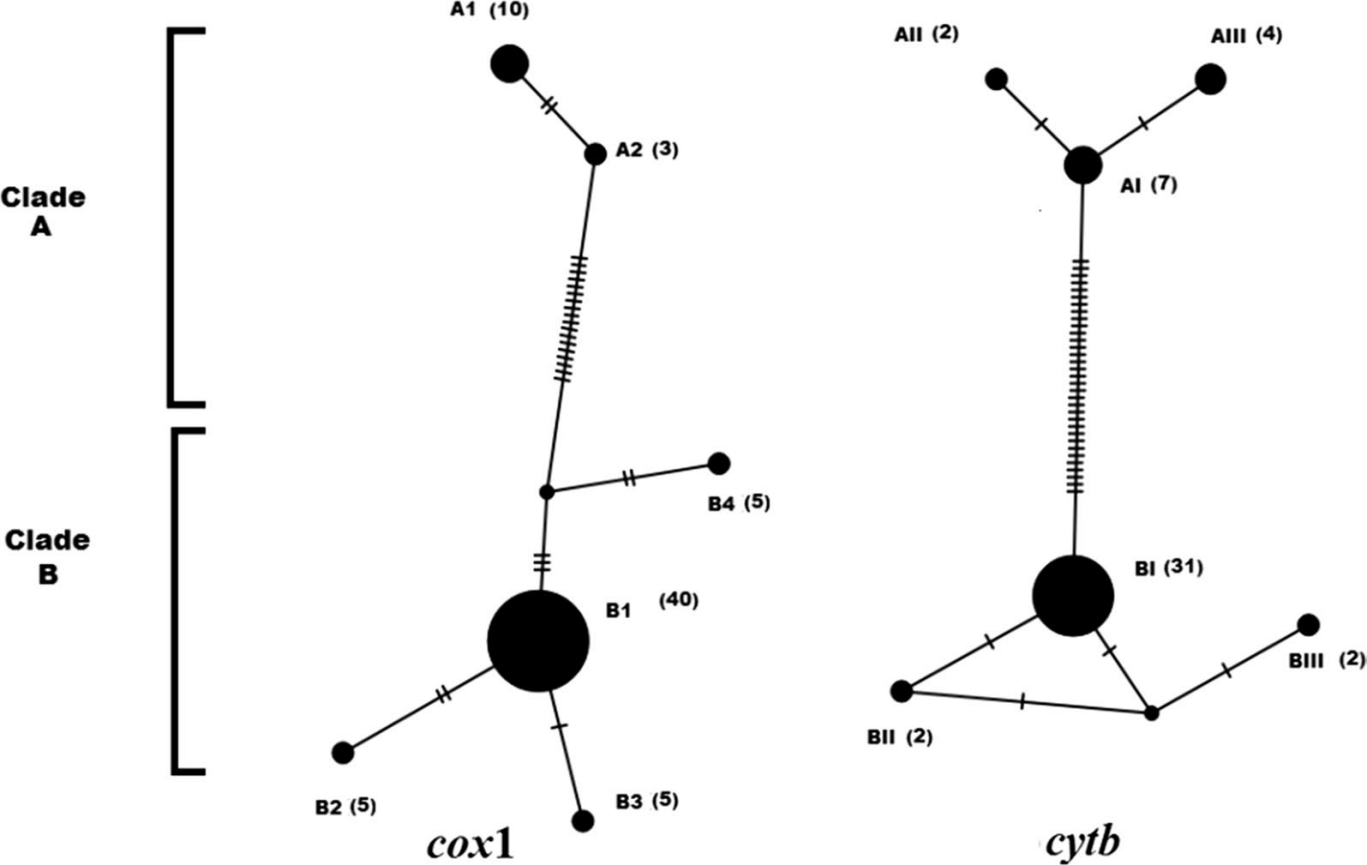
TCS network tree of head lice mitochondrial genes, based on all polymorphic sites. Each dash represents one single nucleotide difference between two neighbouring haplotypes. The numbers in the parentheses after the name of each haplotype denote the number of samples belonging to each haplotype

A 632-bp fragment of the *cytb* gene was generated in 48 randomly (13 samples from each population) selected individuals (Fig. 1, Additional file 1: Figure S1). Of these sequences, 17 representing the population groups were deposited in the GenBank database under the accession numbers MK631767-MK631784. Multiple alignments of these sequences showed 39 SNPs in the amplicons. Analysis of the sequences demonstrated that the studied populations were classified into 6 haplotypes clustered into two groups (ecotypes), A and B. Nucleotide sequence analysis also showed that ecotype B was the dominant form and comprised 73% of the samples. Haplotype diversity and nucleotide diversity in groups A and B were 0.589 and 0.02261, respectively. Analysis of nucleotide sequences in groups A and B indicated that most of the variation sites were in the proximal 3′-region in amplified fragments (Fig. 1). Haplotype BI was the predominant haplotype and was identified in 64.6% of the samples. This haplotype was found in all of the studied areas. Each of the two haplotypes BII and BIII, which differed from haplotype BI in one nucleotide, comprised 8.2% of the total samples and were found in Zanjan district. Haplotype AI which was identified in 14.6% of samples, and the remaining haplotypes (AII and AIII), which varied from haplotype AI by one nucleotide, were identified in 4.1% and 8.3% of the samples, respectively (Fig. 2). Haplotypes AI, AII and AIII which clustered in Clade A were found in rural and suburban areas.

The BLAST analysis of *cytb* sequences revealed that Group B was 100% identical with the Clade B reference strain (American head louse; GenBank: AY696013). Additionally, group A showed 99% similarity with the reference strain of Clade A (GenBank: AY696047).

**Fig. 3:**
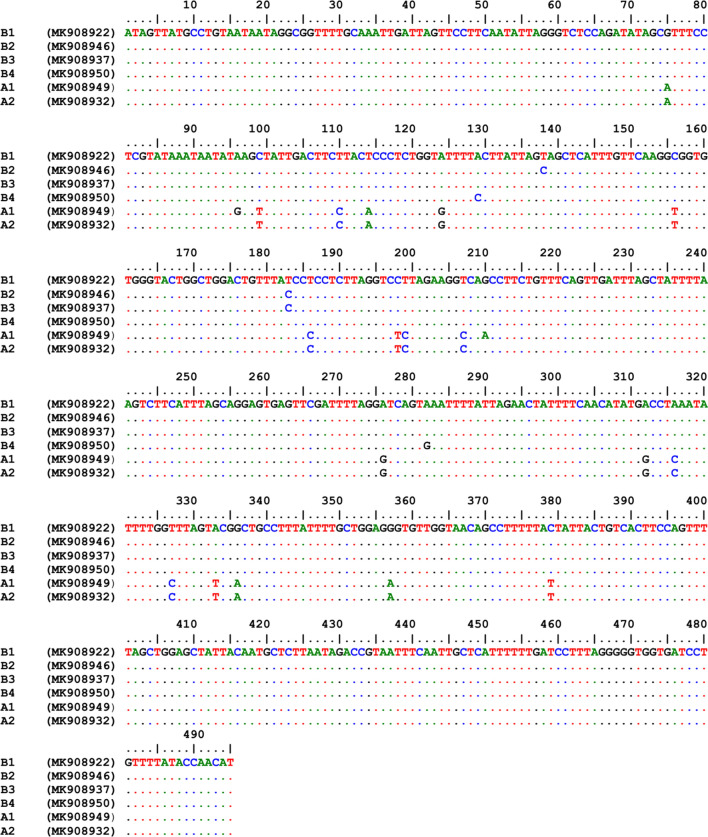
Alignment of the nucleotide sequences of the mitochondrial *cox*1 gene fragment in different haplotypes in head lice populations in the northwest of Iran. The numbers in parentheses are the representative accession numbers of the haplotype groups

Among 68 randomly selected samples from amplicons of the *cox*1 gene subjected to sequencing, a fragment of 495 bp was amplified (Fig. 3, Additional file 2: Figure S2). The nucleotide sequences of 28 amplicons in the studied samples were deposited in GenBank under the accession numbers MK908922–MK908950. Multiple alignment of these samples showed 25 SNP sites. These samples were categorized into 6 haplotypes, and the same as *cytb* groups, they were clustered into two groups, A and B. Haplotype diversity and nucleotide diversity in these samples were 0.424 and 0.01443, respectively. The dominant haplotype was haplotype B1 that included 59% of the samples. This haplotype was found in all the studied areas. Each of the haplotypes B2, B3 and B4 comprised 7.3% of the samples. All four haplotypes (B1, B2, B3 and B4) were merged into Group B. The two remaining haplotypes (A1 and A2), which differed by two nucleotides, included 14.5% and 4.4% of the samples, respectively and assembled in Group A, whose haplotypes were detected in rural and suburban areas. Analysis of nucleotide sequences indicated that ecotype B was the dominant form and comprised 81% of the samples (Fig. 2).

**Fig. 4:**
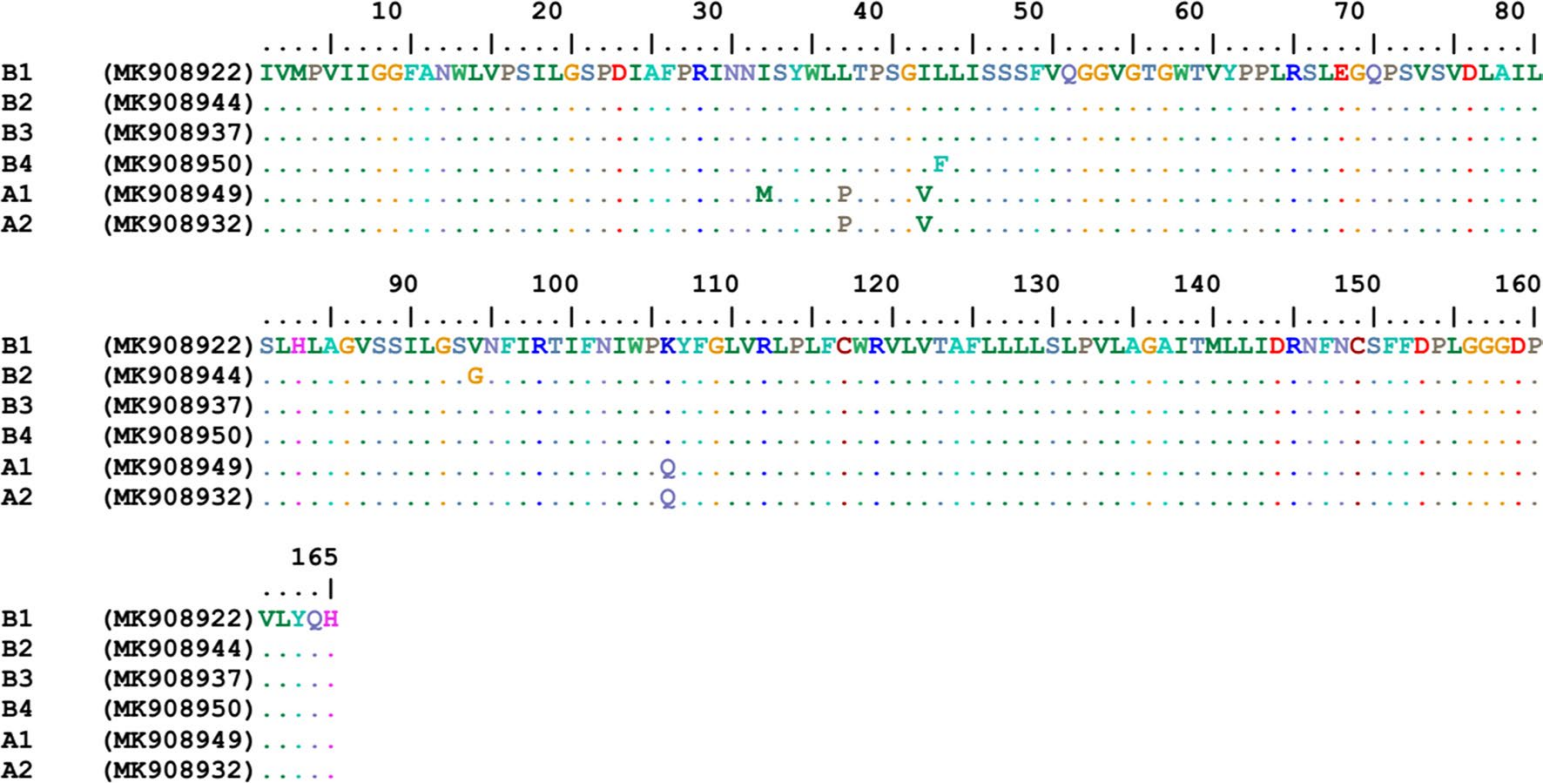
Alignment of the amino acid sequences of the mitochondrial *cox*1 gene fragment in different haplotypes in head lice populations in the northwest of Iran. The numbers in parentheses are the representative accession numbers of the haplotype groups

Based on BLAST analysis, Group A of the studied samples was 100% and 99% identical with the Clade A reference strains, Ottawa *P. humanus capitis* and Iranian *P. humanus humanus* (GenBank: EU93439 and AY589980, respectively). Additionally, Group B exhibited 98% similarity with the Clade B reference strain (American *P. humanus capitis*; GenBank: AY695942). The amplified fragment of the *cox*1 gene encoded 165 amino acids. Translated amino acids in these fragments varied in 6 amino acid sites. Two haplotypes (B2 and B3) differed from the predominant haplotype (B1) in one amino acid. Other differences were the substitution of glycine (G) for valine (V) in haplotype B2 and leucine (L) for phenyl alanine (F) in haplotype B3. The haplotypes A1 and A2 were also dissimilar in one amino acid (substitution of methionine for isoleucine). In the analysis of multiple sequences, variation between groups A and B was observed at 4 sites, and the amino acids P, V, and Q in Group B were replaced with the amino acids L, I, and K in the same group, respectively (Fig. 4).

**Fig. 5:**
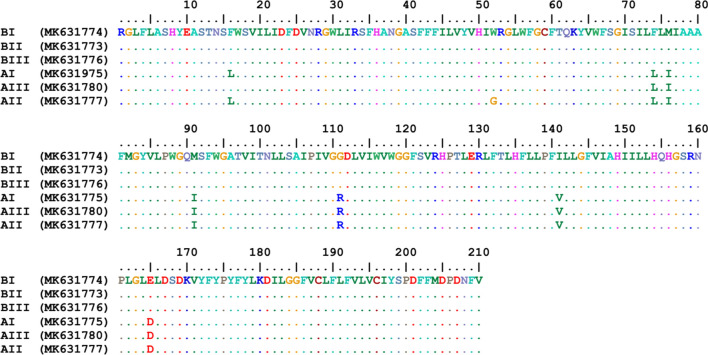
Alignment of the amino acid sequences of the mitochondrial *cytb* gene fragment in different haplotypes in head lice populations in the northwest of Iran. The numbers in parentheses are the representative accession numbers of the haplotype groups

The studied fragments of the *cytb* gene encoded 210 amino acids. Multiple alignment of the amino acid sequences showed 8 polymorphic sites in these fragments. Three haplotypes of Group B were different in two loci, where the amino acid leucine in haplotypes AI and AII was substituted for that of phenyl alanine in haplotype AIII, and the amino acid G in haplotype AII was replaced with the amino acid W in haplotypes AI and AIII. Analysis of the multiple alignment of haplotype sequences in the two groups (A and B) demonstrated 7 polymorphic sites, where the amino acids L, I, I, R, V, and D in Group B were replaced with F, M, M, G, L, and E in Group A, respectively (Fig. 5).

**Fig. 6:**
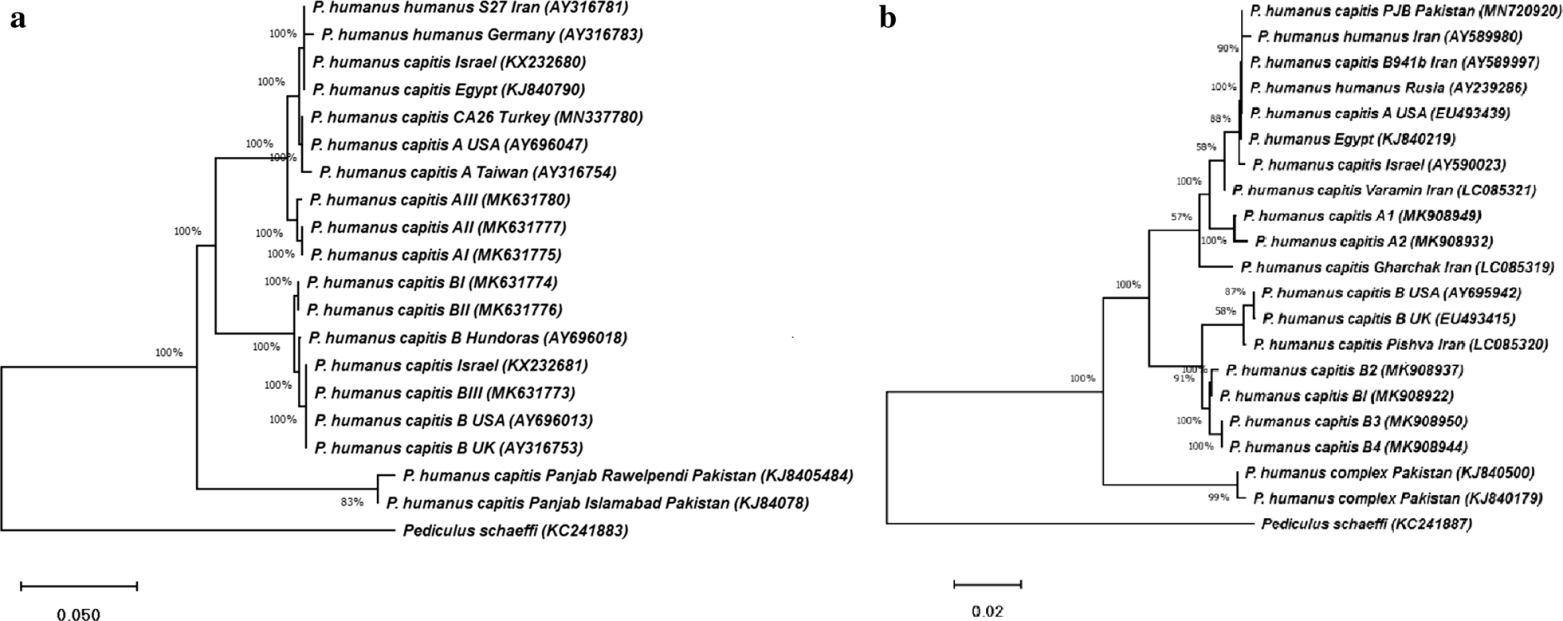
Phylogenetic relationships between different population groups of *P. humanus capitis*. Phylogenetic tree was inferred with Neighbor-Joining and Maximum Likelihood methods in fragments of the *cytb* (**a**) and *cox*1 (**b**) genes in different haplotypes of the present study and their homologues in *P. humanus humanus* and *P*. *humanus capitis* populations. The percentage of replicate trees in which the associated taxa clustered together in the bootstrap test (500 replicates) is shown next to the branches. The scale-bar indicates the Kimura 2-parameter distance

The nucleotide sequences of the present study were combined with 32 sequences, retrieved from GenBank, resulting in a dataset with a total of 20 and 21 sequences for the analysis of *cytb* and *cox*1 fragments, respectively. The Neighbor-Joining and Maximum Likelihood analysis performed for each mtDNA gene consistently recovered two highly divergent and well-supported monophyletic clades (A and B). Phylogenetic analysis indicated that head lice from Varamin (GenBank: LC085321) and Gharchak (GenBank: LC085319) as well as the specimens of body lice (GenBank: AY589980) and head lice (GenBank: AY589997) were merged in Clade A and the head lice reported from Pishva (GenBank: LC085320) was assembled in Clade B. The topology of the phylogenetic tree showed that the populations of head lice in the Middle East were divided into three major clades, A, B and C. The first two clades (A and B) were found in Egypt, Iran, Israel, Pakistan and Turkey, whereas the third clade (clade C) originated only in Pakistan. In the Middle East, the populations of head lice were clustered in Subclade A1, the worldwide subclade, and the American, Chinese and German populations were the sister groups for Clade A. The populations of Clade B in the USA and Europe were the sister group for the haplotypes of Clade B in the Middle East (Fig. 6).

## Discussion

To the best of our knowledge, this is the first study in the Middle East that has widely surveyed field populations of head lice by the molecular analysis of *cox*1 and *cytb* mitochondrial genes. There has previously been no available information on the presence of Clades A and B head lice in the studied areas; therefore, providing relevant molecular data was a high priority for this investigation, with focus on the analysis of head lice taxa and planning control programmes against them in this region.

The present study showed that 8.7% of the girls in various schools in northwestern Iran were infested with pediculosis. Although different levels of infestation have been reported in diverse areas of Iran, the rate of infestation in the areas studied is equivalent to the average rate of pediculosis in the country [31]. In a study by Shayeghi et al. [36], the distribution of pediculosis among primary school students in Khajeh City (East Azerbaijan Province), was 4.4%. In another investigation conducted in Urmia (West Azerbaijan Province), Hazrati Tappeh et al. [37] studied the head lice prevalence among female students and reported a relative frequency of 4%. Two similar surveys recorded 4.5% and 11% frequency of head lice among elementary school students in Asadabad (Hamadan Province) and Zanjan (Zanjan Province) districts, respectively [39, 48]. A previous report from Meshgin Shahr County (Ardabil Province), has also uncovered a high incidence of pediculosis among primary school students [40]. Our results, and those of other researchers related to the prevalence of head lice in studied areas, highlight the fact that head lice have considerable distribution among female students, and its trend is rising. Given the rising prevalence of pediculosis in recent years, reforming policies toward controlling and preventing this ectoparasite is the most important step for authorities to overcome this problem. For the past two decades, application of permethrin in the form of shampoo has been one of the most frequent strategies for pediculosis control in northwestern Iran, the same as elsewhere [49]. Permethrin inefficiency in the treatment of pediculosis appears to contribute to the persistence of this infection. Therefore, future studies are needed to evaluate the level of susceptibility of different populations to permethrin and other common compounds.

Sequence analysis of the *cox*1 and *cytb* fragments showed the guanine and cytosine (GC) content of 38% and 35%, which were relatively comparable with that of other investigations (34.99% and 34.28%), respectively [21, 43]. The low GC content in *cox*1 fragments elevate the probability of basic changes and variations in nucleotide sequences. Since the amount of adenine and thymine (AT) content and nucleotide diversity in the amplified fragment of the *cytb* gene was higher than that of the *cox*1 gene, using this fragment of the *cytb* gene as the main molecular marker is suggested for accurate determination of the genetic variation in lice populations in future. Moreover, due to the variation in the proposed clades close to the 3’-end of the mitochondrial *cytb* fragment, this region is offered as an ideal molecular marker for differentiation of Clades A and B and thus is recommended for designing specific primers for nested-PCR or real-time PCR methods in future investigations.

The molecular analysis of the nucleotide sequences revealed that the studied populations were located in clades A and B. In a previous study in Iran, two mitochondrial groups of head lice were reported from central regions [50]. Comparison of the nucleotide sequence of these groups with the results of the present study indicates the appearance of two clades (A and B) in the central regions of Iran. Findings from studies in Bisha governorate of Saudi Arabia [34], Pakistan [43], Israel and Iran [51], Turkey [52] and Egypt [43] also show the presence of the two mentioned clades in these countries. Our results and findings in the surrounding geographical areas are documentary evidence for the presence of these two clades in the Middle East. This observation is similar to the results of other studies showing that ecotype A has a global distribution [8, 22, 24, 51]. Phylogenetic analysis of Clade A in the present study reflected that this linkage has 99% similarity with Eurasian Subclade A1 and the Colombian Subclade A3. Since Clade A is comprised of both head and body lice, and body lice were proven to be a major vector of louse-borne-pathogens; therefore, lice populations of Clade A in studied areas may transmit many pathogens. Consequently, development of control programmes against populations of this clade seems to be necessary.

Based on the findings of the present work, the studied populations of rural and suburban areas are heterogeneous, and Clades A and B are distributed sympatrically. The simultaneous presence of the two clades in rural areas and suburbs may be due to the lack of selective pressure on these linkage groups. As the impact of socioeconomic factors and the level of education are similar in these areas, the absence of pressure is probably related to inadequate coverage of both pediculosis control programmes and health services. However, in urban areas, due to the availability of health services and over-the-counter pediculicides, there is a selective pressure of insecticides. It seems that the response of the populations to the selective pressures may vary, and the continuous use of insecticides in recent years has homogenized lice populations. Thus, the homogeneity of the Clade B in urban areas may come from the adaptive responses of lice populations to insecticide selection pressure, resistance to insecticides, and failure in control programmes. Accordingly, determining the role of resistance to insecticides in adaptive responses of Clade B populations is of paramount importance that needs to be studied in the future.

To the best of our knowledge, this study is the first to identify the presence of lice of Clade B as a predominant lineage in the northwest of Iran. Phylogenetic analysis of nucleotide sequences also confirmed the presence of lice of clade B in the central region of Iran (Pishva, Tehran Province), Pakistan, Turkey, Israel and Egypt. In spite of the high prevalence of Clade B in the USA [7] and the presence of this clade in nits belonging to the pre-Colombian mummies from Cameron [22], there is a challenge on the acceptance of Central America as an origin site for Clade B, because lice in Central America may have been introduced by the first immigrants from Asia [24]. This ancient clade mostly resulted from a recent host switching, from Neanderthals or Denisovans to modern humans, in Eurasia and diverged from Clade A between 0.7 and 1.2 MYa [21]. Lice infestation of the ancient inhabitants (Sumerian, Akkadian and Egyptian sources) was evidenced in the Middle East, and the oldest head lice remains were recovered from an individual who lived in the Nahal Hemar Cave near the Dead Sea in Israel during the Neolithic era, 9000 years ago. Moreover, recent archaeological excavation near the Dead Sea confirmed Clade B in the remains of ancient head lice and their eggs which dates back about 2000 years [53]. Due to the importance of the Middle East as the main immigration route for modern humans, the high prevalence of Clade B head lice in the Middle East, and its recent discovery among head lice remains from Israel, it is more likely that Clade B was formed in the Middle East. Nonetheless, it is interesting to test a large number of head lice, especially ancient specimens, for detecting the exact origin of this clade.

## Conclusions

To the best of our knowledge, this is the first broad investigation of genetic variation in different head lice populations in the Middle East. The present study found that infection with *P. humanus capitis* is significant in the northwest of Iran. In view of this, development and modification of control programmes are essential to combat this health problem. The head lice populations in the studied area were found to belong to two clades (A and B), with varied geographical distribution patterns. Clade B was found throughout the area, whereas clade A was detected only in suburban and rural areas. The presence of cultural, economic and social factors and different levels of access to health services and pediculicides and sensitivity to permethrin may be implicated in the appearance of various distribution patterns in these clades. The *cytb* gene is suggested as a useful molecular marker for studying the two clades (A and B), and for designing specific primers in order to screen these clades and identify the effect of main epigenetic factors on the abundance of these clades in future studies.

## Supplementary information

**Additional file 1: Figure S1.** Alignment of the nucleotide sequences of the mitochondrial *cytb* gene fragments.

**Additional file 2: Figure S2.** Alignment of the nucleotide sequences of the mitochondrial *cox*1 gene fragments.

## Data Availability

All data generated or analysed during this study are included in this published article and its additional files. The newly generated sequences were submitted to the GenBank database under the accession numbers MK631767-MK631784 (*cytb*) and MK908922-MK908950 (*cox*1).

## Abbreviations

BLAST: Basic Local Alignment Search Tool
*cox*1: cytochrome *c* oxidase subunit 1 gene
*cytb*: cytochrome *b* gene
gDNA: genomic DNA
mtDNA: mitochondrial DNA
MYa: million years ago
PCR: polymerase chain reaction
ZUMS: Zanjan University of Medical Sciences
