# Supplementary material for: Diversity of mitochondrial genes and predominance of Clade B in different head lice populations in the northwest of Iran
Source: Parasit Vectors. 2020 Sep 23;13:485. doi: 10.1186/s13071-020-04364-z (PMC7510113; doi:10.1186/s13071-020-04364-z)
Supplement: Supplementary file 1 — Additional file 1: Figure S1. Alignment of the nucleotide sequences of the mitochondrial cytb gene fragments. [file 13071_2020_4364_MOESM1_ESM.rtf]

Additional file 1: Figure S1. Alignment of the nucleotide sequences of the mitochondrial cytb gene fragment in some of samples of head louse populations in the northwest of Iran. 


  

                    10        20        30        40        50        60        70        80
             ....|....|....|....|....|....|....|....|....|....|....|....|....|....|....|....|
9606G05_PBF  AGAGGCCTTTTTCTAGCTTCTCATTATGAGGCTTCTACTAATTCGTTTTGAAGTGTAATTTTAATTGATTTTGATGTAAA 
9606G07_PBF  ................................................................................ 
9606G11_PBF  ................................................................................ 
9606G21_PBF  ......-......................................................................... 
9606G70_PBF  ................................................................................ 
9606G72_PBF  ................................................................................ 
9606G02_PBR  ................................................................................ 
9606G04_PBR  ................................................................................ 
9606G08_PBR  ................................................................................ 
9606G10_PBR  ................................................................................ 
9606G20_PBR  ................................................................................ 
9606G22_PBR  ................................................................................ 
9606G71_PBR  ................................................................................ 
9606G73_PBR  ................................................................................ 
9606G18_PBR  ................................................................................ 
9606G09_PBF  ................................................................................ 
9606G16_PBR  .....T........G.........................................T....................... 
9606G24_PBR  .....T........G.........................................T....................... 
9606G12_PBR  .....T........G.........................................T....................... 
9606G06_PBR  .....T........G.........................................T....................... 
9606G19_PBR  .....T........G.........................................T....................... 
9606G03_PBR  .....T........G.........................................T....................... 

                      90       100       110       120       130       140       150       160 
             ....|....|....|....|....|....|....|....|....|....|....|....|....|....|....|....|
9606G05_PBF  TAGAGGATGATTGATTCGTAGTTTTCATGCTAATGGCGCTTCTTTTTTCTTTATTCTTGTTTACGTTCATATTTGGCGTG 
9606G07_PBF  ................................................................................ 
9606G11_PBF  ................................................................................ 
9606G21_PBF  ................................................................................ 
9606G70_PBF  ................................................................................ 
9606G72_PBF  ................................................................................ 
9606G02_PBR  ................................................................................ 
9606G04_PBR  ................................................................................ 
9606G08_PBR  ................................................................................ 
9606G10_PBR  ................................................................................ 
9606G20_PBR  ................................................................................ 
9606G22_PBR  ................................................................................ 
9606G71_PBR  ................................................................................ 
9606G73_PBR  ................................................................................ 
9606G18_PBR  ................................................................................ 
9606G09_PBF  ................................................................................ 
9606G16_PBR  ......G..G.......................C.................C........C................... 
9606G24_PBR  ......G..G.......................C.................C........C................... 
9606G12_PBR  ......G..G.......................C.................C........C............G...... 
9606G06_PBR  ......G..G.......................C.................C........C............G...... 
9606G19_PBR  ......G..G.......................C.................C........C................... 
9606G03_PBR  ......G..G.......................C.................C........C................... 

                     170       180       190       200       210       220       230       240 
             ....|....|....|....|....|....|....|....|....|....|....|....|....|....|....|....|
9606G05_PBF  GCTTATGATTTGGTTGTTTTACGCAAAAATATGTTTGATTTTCAGGAATTTCTATTCTTTTTCTTATGATAGCAGCAGCT 
9606G07_PBF  ................................................................................ 
9606G11_PBF  ................................................................................ 
9606G21_PBF  ................................................................................ 
9606G70_PBF  ................................................................................ 
9606G72_PBF  ................................................................................ 
9606G02_PBR  ................................................................................ 
9606G04_PBR  ................................................................................ 
9606G08_PBR  ................................................................................ 
9606G10_PBR  ................................................................................ 
9606G20_PBR  ................................................................................ 
9606G22_PBR  ................................................................................ 
9606G71_PBR  ................................................................................ 
9606G73_PBR  ................................................................................ 
9606G18_PBR  ................................................................................ 
9606G09_PBF  ................................................................................ 
9606G16_PBR  .T....................A....................................C.......A............ 
9606G24_PBR  .T....................A....................................C.......A............ 
9606G12_PBR  .T....................A....................................C.......A............ 
9606G06_PBR  .T....................A....................................C.......A............ 
9606G19_PBR  .T....................A....................................C.......A............ 
9606G03_PBR  .T....................A....................................C.......A............ 

                     250       260       270       280       290       300       310       320  
             ....|....|....|....|....|....|....|....|....|....|....|....|....|....|....|....|
9606G05_PBF  TTTATGGGGTATGTTCTACCTTGAGGTCAAATGTCTTTTTGAGGGGCGACTGTAATTACTAATCTTTTAAGTGCTATTCC 
9606G07_PBF  ................................................................................ 
9606G11_PBF  ................................................................................ 
9606G21_PBF  ................................................................................ 
9606G70_PBF  ................................................................................ 
9606G72_PBF  ................................................................................ 
9606G02_PBR  ................................................................................ 
9606G04_PBR  ................................................................................ 
9606G08_PBR  ................................................................................ 
9606G10_PBR  ................................................................................ 
9606G20_PBR  ................................................................................ 
9606G22_PBR  ................................................................................ 
9606G71_PBR  ................................................................................ 
9606G73_PBR  ................................................................................ 
9606G18_PBR  ................................................................................ 
9606G09_PBF  ................................................................................ 
9606G16_PBR  .................T..............A...........A................................... 
9606G24_PBR  .................T..............A...........A................................... 
9606G12_PBR  .................T..............A...........A................................... 
9606G06_PBR  .................T..............A...........A................................... 
9606G19_PBR  .................T..............A...........A................................... 
9606G03_PBR  .................T..............A...........A................................... 

                     330       340       350       360       370       380       390       400 
             ....|....|....|....|....|....|....|....|....|....|....|....|....|....|....|....|
9606G05_PBF  TATTGTTGGAGGAGATTTGGTTATTTGAGTGTGAGGAGGGTTTTCAGTTAGACATCCTACTTTAGAGCGATTGTTTACCC 
9606G07_PBF  ................................................................................ 
9606G11_PBF  ................................................................................ 
9606G21_PBF  ................................................................................ 
9606G70_PBF  ................................................................................ 
9606G72_PBF  ................................................................................ 
9606G02_PBR  ................................................................................ 
9606G04_PBR  ................................................................................ 
9606G08_PBR  ................................................................................ 
9606G10_PBR  ................................................................................ 
9606G20_PBR  ................................................................................ 
9606G22_PBR  ................................................................................ 
9606G71_PBR  ................................................................................ 
9606G73_PBR  ................................................................................ 
9606G18_PBR  ................................................................................ 
9606G09_PBF  ................................................................................ 
9606G16_PBR  ..........A..........................................................G........T. 
9606G24_PBR  ..........A..........................................................G........T. 
9606G12_PBR  ..........A..........................................................G........T. 
9606G06_PBR  ..........A..........................................................G........T. 
9606G19_PBR  ..........A..........................................................GC.......T. 
9606G03_PBR  ..........A..........................................................G........T. 

                     410       420       430       440       450       460       470       480 
             ....|....|....|....|....|....|....|....|....|....|....|....|....|....|....|....|
9606G05_PBF  TTCACTTTCTTTTACCGTTTATCTTGTTGGGATTTGTTATAGCTCACATTATTCTTCTTCATCAACACGGCTCTAGAAAT 
9606G07_PBF  ................................................................................ 
9606G11_PBF  ................................................................................ 
9606G21_PBF  ................................................................................ 
9606G70_PBF  ................................................................................ 
9606G72_PBF  ................................................................................ 
9606G02_PBR  ................................................................................ 
9606G04_PBR  ................................................................................ 
9606G08_PBR  ................................................................................ 
9606G10_PBR  ................................................................................ 
9606G20_PBR  ................................................................................ 
9606G22_PBR  ................................................................................ 
9606G71_PBR  ................................................................................ 
9606G73_PBR  ................................................................................ 
9606G18_PBR  ................................................................................ 
9606G09_PBF  ................................................................................ 
9606G16_PBR  ....................G....A.....G.......................C..C..C........T......... 
9606G24_PBR  ....................G....A.....G.......................C..C..C........T......... 
9606G12_PBR  ....................G....A.....G.......................C..C..C........T......... 
9606G06_PBR  ....................G....A.....G.......................C..C..C........T......... 
9606G19_PBR  ....................G....A.....G.......................C..C..C........T......... 
9606G03_PBR  ....................G....A.....G.......................C..C..C........T......... 

                     490       500       510       520       530       540       550       560  
             ....|....|....|....|....|....|....|....|....|....|....|....|....|....|....|....|
9606G05_PBF  CCTTTGGGGTTGGAGCTGGATAGTGATAAGGTTTATTTTTATCCTTATTTTTATCTAAAAGATATTTTAGGAGTTTTTGT 
9606G07_PBF  ................................................................................ 
9606G11_PBF  ................................................................................ 
9606G21_PBF  ................................................................................ 
9606G70_PBF  ................................................................................ 
9606G72_PBF  ................................................................................ 
9606G02_PBR  ................................................................................ 
9606G04_PBR  ................................................................................ 
9606G08_PBR  ................................................................................ 
9606G10_PBR  ................................................................................ 
9606G20_PBR  ................................................................................ 
9606G22_PBR  ................................................................................ 
9606G71_PBR  ................................................................................ 
9606G73_PBR  ................................................................................ 
9606G18_PBR  ................................................................................ 
9606G09_PBF  ................................................................................ 
9606G16_PBR  .....A..A.....TT.............A.................C.........................G...... 
9606G24_PBR  .....A..A.....TT.............A.................C.........................G...... 
9606G12_PBR  .....A..A.....TT.............A.................C.........................G...... 
9606G06_PBR  .....A..A.....TT.............A.................C.........................G...... 
9606G19_PBR  .....A..A.....TT.............A.................C.........................G...... 
9606G03_PBR  .....A..A.....TT.............A.................C.........................G...... 

                     570       580       590       600       610       620       630      
             ....|....|....|....|....|....|....|....|....|....|....|....|....|....|.
9606G05_PBF  GTGTTTGTTTTTATTTGTTTTGGTCTGCATTTATTCGCCGGACTTCTTTATGGACCTATTTGTTTTGGTCT 
9606G07_PBF  ....................................................................... 
9606G11_PBF  ....................................................................... 
9606G21_PBF  .................................................C..................... 
9606G70_PBF  ....................................................................... 
9606G72_PBF  ....................................................................... 
9606G02_PBR  ....................................................................... 
9606G04_PBR  ....................................................................... 
9606G08_PBR  ....................................................................... 
9606G10_PBR  ....................................................................... 
9606G20_PBR  ....................................................................... 
9606G22_PBR  ....................................................................... 
9606G71_PBR  ....................................................................... 
9606G73_PBR  ....................................................................... 
9606G18_PBR  ....................................................................... 
9606G09_PBF  .......................................A.........G..................... 
9606G16_PBR  ......A................................................................ 
9606G24_PBR  ......A................................................................ 
9606G12_PBR  ......A................................................................ 
9606G06_PBR  ......A................................................................ 
9606G19_PBR  ......A................................................................ 
9606G03_PBR  ......A................................................................ 
