# Supplementary material for: Diversity of mitochondrial genes and predominance of Clade B in different head lice populations in the northwest of Iran
Source: Parasit Vectors. 2020 Sep 23;13:485. doi: 10.1186/s13071-020-04364-z (PMC7510113; doi:10.1186/s13071-020-04364-z)
Supplement: Supplementary file 2 — Additional file 2: Figure S2. Alignment of the nucleotide sequences of the mitochondrial cox1 gene fragments. [file 13071_2020_4364_MOESM2_ESM.rtf]

Additional file 2: Figure S2. Alignment of the nucleotide sequences of the mitochondrial cox1 gene fragment in some of samples of head louse populations in the northwest of Iran. 


                      10        20        30        40        50        60        70        80 
             ....|....|....|....|....|....|....|....|....|....|....|....|....|....|....|....|
9606G25_PCF  ATAGTTATGCCTGTAATAATAGGCGGTTTTGCAAATTGATTAGTTCCTTCAATATTAGGGTCTCCAGATATAGCGTTTCC 
9606G27_PCF  ................................................................................ 
9606G29_PCF  ................................................................................ 
9606G31_PCF  ................................................................................ 
9606G33_PCF  ................................................................................ 
9606G37_PCF  ................................................................................ 
9606G39_PCF  ................................................................................ 
9606G41_PCF  ................................................................................ 
9606G43_PCF  ................................................................................ 
9606G45_PCF  ................................................................................ 
9606G47_PCF  ................................................................................ 
9606G74_PCF  ................................................................................ 
9606G76_PCF  ................................................................................ 
9606G26_PCR  ................................................................................ 
9606G30_PCR  ................................................................................ 
9606G32_PCR  ................................................................................ 
9606G34_PCR  ................................................................................ 
9606G38_PCR  ................................................................................ 
9606G40_PCR  ................................................................................ 
9606G42_PCR  ................................................................................ 
9606G44_PCR  ................................................................................ 
9606G46_PCR  ................................................................................ 
9606G48_PCR  ................................................................................ 
9606G75_PCR  ................................................................................ 
9606G74_PCF  ................................................................................ 
9606G28_PCF  ..........................................................................A..... 
9606G36_PCF  ..........................................................................A..... 
9606G77_PCF  ..........................................................................A..... 
9606G35_PCF  ..........................................................................A..... 

                      90       100       110       120       130       140       150       160 
             ....|....|....|....|....|....|....|....|....|....|....|....|....|....|....|....|
9606G25_PCF  TCGTATAAATAATATAAGCTATTGACTTCTTACTCCCTCTGGTATTTTACTTATTAGTAGCTCATTTGTTCAAGGCGGTG 
9606G27_PCF  ................................................................................ 
9606G29_PCF  ................................................................................ 
9606G31_PCF  ................................................................................ 
9606G33_PCF  ................................................................................ 
9606G37_PCF  ................................................................................ 
9606G39_PCF  ................................................................................ 
9606G41_PCF  ................................................................................ 
9606G43_PCF  ................................................................................ 
9606G45_PCF  ................................................................................ 
9606G47_PCF  ................................................................................ 
9606G74_PCF  .........................................................C...................... 
9606G76_PCF  ................................................................................ 
9606G26_PCR  ................................................................................ 
9606G30_PCR  ................................................................................ 
9606G32_PCR  ................................................................................ 
9606G34_PCR  ................................................................................ 
9606G38_PCR  ................................................................................ 
9606G40_PCR  ................................................................................ 
9606G42_PCR  ................................................................................ 
9606G44_PCR  ................................................................................ 
9606G46_PCR  ................................................................................ 
9606G48_PCR  ................................................................................ 
9606G75_PCR  ................................................................................ 
9606G74_PCF  ................................................C............................... 
9606G28_PCF  ...............G..T..........C...A.........G...............................T.... 
9606G36_PCF  ...............G..T..........C...A.........G...............................T.... 
9606G77_PCF  ...............G..T..........C...A.........G...............................T.... 
9606G35_PCF  ..................T..........C...A.........G...............................T.... 

                     170       180       190       200       210       220       230       240 
             ....|....|....|....|....|....|....|....|....|....|....|....|....|....|....|....|
9606G25_PCF  TGGGTACTGGCTGGACTGTTTATCCTCCTCTTAGGTCCTTAGAAGGTCAGCCTTCTGTTTCAGTTGATTTAGCTATTTTA 
9606G27_PCF  ................................................................................ 
9606G29_PCF  ................................................................................ 
9606G31_PCF  ................................................................................ 
9606G33_PCF  ................................................................................ 
9606G37_PCF  ................................................................................ 
9606G39_PCF  ................................................................................ 
9606G41_PCF  ................................................................................ 
9606G43_PCF  ................................................................................ 
9606G45_PCF  ................................................................................ 
9606G47_PCF  ................................................................................ 
9606G74_PCF  ................................................................................ 
9606G76_PCF  ................................................................................ 
9606G26_PCR  ................................................................................ 
9606G30_PCR  ................................................................................ 
9606G32_PCR  ................................................................................ 
9606G34_PCR  ................................................................................ 
9606G38_PCR  ................................................................................ 
9606G40_PCR  ......................C......................................................... 
9606G42_PCR  ................................................................................ 
9606G44_PCR  ................................................................................ 
9606G46_PCR  ................................................................................ 
9606G48_PCR  ................................................................................ 
9606G75_PCR  ................................................................................ 
9606G74_PCF  ................................................................................ 
9606G28_PCF  .........................C...........TC.......C..A.............................. 
9606G36_PCF  .........................C...........TC.......C..A.............................. 
9606G77_PCF  .........................C...........TC.......C..A.............................. 
9606G35_PCF  .........................C...........TC.......C................................. 

                     250       260       270       280       290       300       310       320 
             ....|....|....|....|....|....|....|....|....|....|....|....|....|....|....|....|
9606G25_PCF  AGTCTTCATTTAGCAGGAGTGAGTTCGATTTTAGGATCAGTAAATTTTATTAGAACTATTTTCAACATATGACCTAAATA 
9606G27_PCF  ................................................................................ 
9606G29_PCF  ................................................................................ 
9606G31_PCF  ................................................................................ 
9606G33_PCF  ................................................................................ 
9606G37_PCF  ................................................................................ 
9606G39_PCF  ................................................................................ 
9606G41_PCF  ................................................................................ 
9606G43_PCF  ................................................................................ 
9606G45_PCF  ................................................................................ 
9606G47_PCF  ................................................................................ 
9606G74_PCF  ........................................G....................................... 
9606G76_PCF  ................................................................................ 
9606G26_PCR  ................................................................................ 
9606G30_PCR  ................................................................................ 
9606G32_PCR  ................................................................................ 
9606G34_PCR  ................................................................................ 
9606G38_PCR  ................................................................................ 
9606G40_PCR  ................................................................................ 
9606G42_PCR  ................................................................................ 
9606G44_PCR  ................................................................................ 
9606G46_PCR  ................................................................................ 
9606G48_PCR  ................................................................................ 
9606G75_PCR  ................................................................................ 
9606G74_PCF  .........................................G...................................... 
9606G28_PCF  ...................................G...................................G...C.... 
9606G36_PCF  ...................................G...................................G...C.... 
9606G77_PCF  ...................................G...................................G...C.... 
9606G35_PCF  ...................................G...................................G...C.... 

                     330       340       350       360       370       380       390       400  
             ....|....|....|....|....|....|....|....|....|....|....|....|....|....|....|....|
9606G25_PCF  TTTGGTTTAGTACGGCTGCCTTTATTTTGCTGGAGGGTGTTGGTAACAGCCTTTTTACTATTACTGTCACTTCCAGTTTT 
9606G27_PCF  ................................................................................ 
9606G29_PCF  ................................................................................ 
9606G31_PCF  ................................................................................ 
9606G33_PCF  ................................................................................ 
9606G37_PCF  ................................................................................ 
9606G39_PCF  ................................................................................ 
9606G41_PCF  ................................................................................ 
9606G43_PCF  ................................................................................ 
9606G45_PCF  ................................................................................ 
9606G47_PCF  ................................................................................ 
9606G74_PCF  ................................................................................ 
9606G76_PCF  ................................................................................ 
9606G26_PCR  ................................................................................ 
9606G30_PCR  ................................................................................ 
9606G32_PCR  ................................................................................ 
9606G34_PCR  ................................................................................ 
9606G38_PCR  ................................................................................ 
9606G40_PCR  ................................................................................ 
9606G42_PCR  ................................................................................ 
9606G44_PCR  ................................................................................ 
9606G46_PCR  ................................................................................ 
9606G48_PCR  ................................................................................ 
9606G75_PCR  ................................................................................ 
9606G74_PCF  ................................................................................ 
9606G28_PCF  .....C.....T..A....................A.....................T...................... 
9606G36_PCF  .....C.....T..A....................A.....................T...................... 
9606G77_PCF  .....C.....T..A....................A.....................T...................... 
9606G35_PCF  .....C.....T..A....................A.....................T...................... 

                     410       420       430       440       450       460       470       480 
             ....|....|....|....|....|....|....|....|....|....|....|....|....|....|....|....|
9606G25_PCF  AGCTGGAGCTATTACAATGCTCTTAATAGACCGTAACTTCAATTGCTCGTTTTTTGACCCTTTAGGGGGTGGTGATCCTG 
9606G27_PCF  ................................................................................ 
9606G29_PCF  ................................................................................ 
9606G31_PCF  ................................................................................ 
9606G33_PCF  ................................................................................ 
9606G37_PCF  ................................................................................ 
9606G39_PCF  ................................................................................ 
9606G41_PCF  ................................................................................ 
9606G43_PCF  ................................................................................ 
9606G45_PCF  ................................................................................ 
9606G47_PCF  ................................................................................ 
9606G74_PCF  ................................................................................ 
9606G76_PCF  ................................................................................ 
9606G26_PCR  ................................................................................ 
9606G30_PCR  ................................................................................ 
9606G32_PCR  ................................................................................ 
9606G34_PCR  ................................................................................ 
9606G38_PCR  ................................................................................ 
9606G40_PCR  ................................................................................ 
9606G42_PCR  ................................................................................ 
9606G44_PCR  ................................................................................ 
9606G46_PCR  ................................................................................ 
9606G48_PCR  ................................................................................ 
9606G75_PCR  ................................................................................ 
9606G74_PCF  ....................................T...........A........T...................... 
9606G28_PCF  ....................................T...........A........T...................... 
9606G36_PCF  ....................................T...........A........T...................... 
9606G77_PCF  ....................................T...........A........T...................... 
9606G35_PCF  ....................................T...........A........T...................... 

                     490 
             ....|....|....
9606G25_PCF  TTTTATACCAACAT 
9606G27_PCF  .............. 
9606G29_PCF  .............. 
9606G31_PCF  .............. 
9606G33_PCF  .............. 
9606G37_PCF  .............. 
9606G39_PCF  .............. 
9606G41_PCF  .............. 
9606G43_PCF  .............. 
9606G45_PCF  .............. 
9606G47_PCF  .............. 
9606G74_PCF  .............. 
9606G76_PCF  .............. 
9606G26_PCR  .............. 
9606G30_PCR  .............. 
9606G32_PCR  .............. 
9606G34_PCR  .............. 
9606G38_PCR  .............. 
9606G40_PCR  .............. 
9606G42_PCR  .............. 
9606G44_PCR  .............. 
9606G46_PCR  .............. 
9606G48_PCR  .............. 
9606G75_PCR  .............. 
9606G74_PCF  .............. 
9606G28_PCF  .............. 
9606G36_PCF  .............. 
9606G77_PCF  .............. 
9606G35_PCF  .............. 
